# Enhancing Bioremediation Potential of *Pseudomonas putida* by Developing Its Acid Stress Tolerance With Glutamate Decarboxylase Dependent System and Global Regulator of Extreme Radiation Resistance

**DOI:** 10.3389/fmicb.2019.02033

**Published:** 2019-09-04

**Authors:** Zikang Zhou, Yuping Liu, Giulio Zanaroli, Zhiqiang Wang, Ping Xu, Hongzhi Tang

**Affiliations:** ^1^State Key Laboratory of Microbial Metabolism, School of Life Sciences and Biotechnology, Shanghai Jiao Tong University, Shanghai, China; ^2^Joint International Research Laboratory of Metabolic and Developmental Sciences, Shanghai Jiao Tong University, Shanghai, China; ^3^Department of Civil, Chemical, Environmental and Materials Engineering – DICAM, University of Bologna, Bologna, Italy; ^4^SUS Environment Co., Ltd., Shanghai, China

**Keywords:** acid resistance, bioremediation, glutamate decarboxylase (GAD) system, *gadBC*, global regulator, IrrE

## Abstract

The extensive use of acids in a variety of manufacturing industries results in the increase of discharged acidic waste stream into the environment. Such co-pollution of acids and other organic pollutants limits the biodegradation capability of neutrophilic degraders. With high-throughput genetic techniques, we aim to improve the acid tolerance of a pollutant-degrading bacterium, *Pseudomonas putida* S16 by genetically engineering it with the glutamate decarboxylase (GAD)-dependent system and the global regulator (IrrE) of extreme radiation resistance. The engineered strains holding either GAD system or irrE regulator could grow under pH 4.5, compared to the wild type. They could also degrade over 90% of a selected pollutant (benzoate or nicotine) under pH 5.0 in 48 h, while no biodegradation was detected with the wild type under the same conditions. We conclude that acid stress tolerance by the possession of the GAD system or IrrE regulator in pollutant-degrading bacteria would be a promising approach to enhance their viability and biodegrading activities in bioremediation of acidic wastes.

## Introduction

Co-pollution between acids and organic wastes often occurs in the effluents discharged from agriculture and manufacturing industries, and persistence of such pollutants are harmful to any living organisms (Kjeldsen et al., 2002; Foo and Hameed, 2009). In food industries, benzoate is a typical aromatic pollutant found in the waste stream at typical pH 4.0 (Oie et al., 2007; Xie et al., 2009). Another example is nicotine, which is a predominant compound contaminated in the effluent from tobacco factories. These pollutants usually exist in the acidic waste stream at shallow pH values (pH < 5) (Dixon et al., 2000; Zhong et al., 2010; Li et al., 2019). These acidic conditions limit the survival and biodegradation activities of neutrophilic pollutant-degrading bacteria like pseudomonads that generally dominate the polluted sites. Genetics improvement of these bacteria by enhancing their acid stress resistance would be a promising strategy to optimize biological waste treatments.

Some bacteria have evolved various acid-resistance mechanisms, such as the glutamate decarboxylase (GAD)-dependent system, F1-F0-ATPase proton pump, and protection or repair of macromolecules (Su et al., 2011; De Biase and Pennacchietti, 2012). The GAD-dependent system exists in various bacteria, and it involves one or two glutamate decarboxylases (GadA/B) and one antiporter, GadC (Ma et al., 2012; Kanjee and Houry, 2013). GadA/B catalyzes the conversion of protonated glutamate to γ-aminobutyric acid (GABA), and GadC exports GABA in exchange for a new extracellular glutamate molecule and consuming intracellular protons. *Deinococcus radiodurans* has a global regulator, IrrE, which plays a vital role in the protection of this bacterium against radiation (Lin et al., 2013). This IrrE regulator also involves diverse resistance mechanisms of *Escherichia coli* toward osmotic pressure, oxidative stress, ethanol, butanol, acetate, and inorganic acid (Gao et al., 2003; Pan et al., 2009; Chen et al., 2011; Ma et al., 2011).

Previous studies revealed that *Pseudomonas putida* S16 is a non-pathogenic member of the genus *Pseudomonas*, and utilizes a variety of organic wastes as a sole source of carbon and/or nitrogen (Wang et al., 2004, 2007). For instance, *P. putida* S16 effectively degraded nicotine through the pyrrolidine pathway (Tang et al., 2013; Hu et al., 2019). However, this bacterium cannot grow well under acidic conditions (optimal pH7.5), which depletes its value in bioremediation of acidic wastes. The lack of acid stress tolerance of *P. putida* S16 was subsequently confirmed with the absence of genes that play crucial roles in the GAD system (*gadBC*) and the IrrE regulator (*irrE*) in its genome (Yu et al., 2011).

In this study, we employed synthetic biology strategies to improve the acid stress tolerance of a pollutant-degrading bacterium, *P. putida* S16 by cloning with the expressing plasmid of the GAD system or the IrrE regulator. Viability, growth, and pollutant-degrading activities of the engineered strains under acidic conditions were tested in comparison with the wild type. A typical set of organic pollutants found co-polluting in the acidic waste stream produced by industries in China, i.e., nicotine and benzoate were used in the biodegradation experiments. The possibility and benefits of using genetically modified microbes to optimize bioremediation technology were addressed and discussed in this work.

## Materials and Methods

### Materials

L-Nicotine (purity, ≥99%) was purchased from Fluka Chemie GmbH (Buchs Corp., Switzerland). Restriction enzymes, *Nco*I and *Xho*I were purchased from TaKaRa (Dalian, China). Sodium benzoate and glycerin were purchased from Sinopharm Chemical Reagent Co., Ltd. Nucleotide sequencing service was provided by BioSune Company (Shanghai, China).

### Bacterial Strains and Growth Conditions

*P. putida* S16 was grown in Luria-Bertani (LB) broth (composition per L: 5 g Yeast extract, 10 g Typtone, 10 g NaCl) at 30°C or in mineral salt medium (MSM, composition per L: 13.3 g K_2_HPO_4_⋅3H_2_O, 4 g KH_2_PO_4_, 0.2 g MgSO_4_⋅7H_2_O, and 0.5 mL trace elements solution) supplemented with 0.1% (w/v) L-nicotine as for the nicotine medium pH5.5 or 7 (Tang et al., 2013) or 1% (w/v) glycerin and 0.1% (w/v) ammonium sulfate as for glycerin medium pH4, 4.5, and 5. The supplemented compounds served as the sole source of carbon, nitrogen, and energy. The trace elements solution contained 0.1 g ZnSO_4_, 0.1 g Na_2_MoO_4_⋅2H_2_O, 0.05 g CuCl_2_⋅2H_2_O, 0.05 g CaCl_2_⋅2H_2_O, 0.004 g FeSO_4_⋅7H_2_O, 0.008 g MnSO_4_⋅H_2_O, and 0.05 g Na_2_WO_4_⋅2H_2_O per 1 L of 0.1 M HCl.

*E. coli* BL21(DE3) was the source of the GAD system, which was grown in LB broth or agar (solidified with 1.5% (w/v) agar powder to the liquid medium) at 37°C. *D. radiodurans* R1 (ATCC 13939) served as the source of the IrrE regulator, which was cultivated in tryptone-glucose-yeast extract (TGY) broth or agar, containing 0.5% tryptone, 0.3% yeast extract, and 0.1% glucose, and 1.5% agar powder for the agar medium. The liquid culture of any bacteria was incubated with shaking at 200 rpm (30 mg/mL tetracycline might be added to the media if necessary).

### Construction of Acid Stress Tolerance Bacteria

The genomic DNA templates of *E. coli* BL21(DE3) and *D. radiodurans* R1 were extracted by using Wizard Genomic DNA Purification Kit (PROMEGA, A1125). Following, the target gene expressing the GAD system was the *gadBC* gene, which was amplified by PCR using a template from the genomic DNA of *E. coli* BL21 with primers: 5′-CCGCCATGGGATAATTCAGGAGGCACAGAA-3′ and 5′ -GTGCTCGAGTTAGTGTTTCTTGTCATTCAT-3′. For the IrrE regulator, *irrE* gene and the GroESL promoter from the genomic DNA of *D. radiodurans* R1 was extracted and used as the template. The PCR primers were 5′-AGGCGACCGCGATGTGCCCAGTGCCAA-3′ and 5′-GTGCT CGAGTCCAGTTCACTGTGCAGC-3′ for the *irrE* gene; and 5′-CCGCCATGGGGATACCCCCATTCCCCG-3′ and 5′-AC TGGGCACATCGCGGTCGCCTAAAGG-3′ for the GroESL promoter. To generate the DNA sequence of GroESL-*irrE*, the recombinant PCR was then performed using EasyGeno Assembly Cloning Kit (TIANGEN, VI1201) with primers 5′-GTGCTCGAGTCCAGTTCACTGTGCAGC-3′ and 5′- CCG CCATGGGGATACCCCCATTCCCCG-3′.

The PCR products were firstly double digested with *Nco*I and *Xho*I, and then ligated into the *Nco*I and *Xho*I sites of plasmid pME6032 (Tang et al., 2013). The recombinant plasmid pME-Gad or pME-GirrE was introduced into the competent cells of *P. putida* S16 (Tang et al., 2013) by electroporation with a field strength of 125,000 V/cm, the electric resistance of 200 Ω, and a time constant of ∼5.0 ms. The original plasmid pME6032 served as the control. All products were cultured on LB selective plates with tetracycline after electroporation. The positive clones picked from the plates and sent for sequencing.

### Growth Assays at Different pH Values

Viability and growth of the engineered bacteria, *P. putida* S16 harboring the recombinant plasmid pME-Gad or pME-GirrE were tested under acidic conditions compared with the wildtype holding the plasmid pME6032 as a control. The assays were carried out by growing each bacterial strain in MSM supplemented with 10 g/L glycerin (as a carbon source) and 1 g/L (NH_4_)_2_SO_4_ (as a nitrogen source) at different pH values of 4.0, 4.5, and 5.0. The pH was adjusted with 88 mM H_3_PO_4_. Growth conditions were monitored in the Bioscreen C MBR (Finland) at 30°C, with absorbance at 600 nm, and shaking continuously at a medium speed. The experimental set was independently prepared and measured in triplicate.

### Biodegradation Experiments

The biodegradation assay was carried out under acidic conditions at pH 5.5 in comparison with the neutral conditions (pH 7). Either engineered or wildtype strain of *P. putida* S16 was grown in sodium benzoate medium (MSM plus 1 g/L sodium benzoate and 1 g/L ammonium sulfate) at 30°C with shaking at 200 rpm incubator. Equal portions of the bacterial cultures were sampled, which were centrifuged at 12,000 × *g* to remove precipitates. The supernatant was filtered with 0.22 μm membrane filter (PALL) before measuring the UV spectra and the concentration of sodium benzoate by high-performance liquid chromatography (HPLC). The HPLC system was an Eclipse XDB-C18 column (column size: 4.6 mm × 250 mm; particle size, 5 μm; Agilent), and the detection wavelength of the UV detector was 259 nm. A mixture of acetonitrile and 1 mM H_2_SO_4_ (15:85, v/v) served as the mobile phase, with a flow rate of 0.5 mL/min at 30°C. Qualitative and quantitative analysis of sodium benzoate relied on the retention time and peak areas of the samples in comparison with the known concentrations of standards (Supplementary Figure S2).

To test the efficiency of nicotine degradation, either engineered or wild-type strains of *P. putida* S16 were cultivated separately in the nicotine medium pH 7.0 and pH 5.5 at 30°C. The culture broth was sampled during cultivation, in which bacterial cells were removed by centrifugation at 12,000 × *g* and 4°C for 5 min. The supernatant was used for measuring ultraviolet (UV) absorption and HPLC analysis. The supernatant was diluted with 0.1 M HCl and scanned with UV2550 (SHIMADZU) spectrophotometer to record the UV spectrogram. Qualitative and quantitative analysis of nicotine relied on the retention time and peak areas of the samples in comparison with the known concentrations of standards (Supplementary Figure S3).

### Evaluation of Gene Expression by Reverse Transcription-Quantitative PCR (RT-qPCR)

Experiments in this section were performed routinely in triplicate with control and nicotine induction cultures of *P. putida* S16 harboring pME6032, pME-Gad, or pME-GirrE. A single colony of recombinants or control was picked randomly from MSM plate, and a 1:100 dilution of a fresh overnight culture was inoculated in three 250-mL flasks containing 50 mL MSM (control) or MSM plus 1 g/L nicotine (induction).

Batch cultures were incubated at 30°C with shaking (200 rpm) to early-exponential phase (OD_600_ ∼0.5). The early-exponential cells were harvested by centrifugation at 14,000 × *g* for 2 min. Total RNA was extracted from ∼1 × 10^8^ cells using an RNAprep pure cell/bacteria kit (Tiangen), and quantified by NanoDrop (Thermo Fisher Scientific). Then, total RNA was treated by 0.8 mg of DNase (Fermentas), and reverse transcribed to cDNA using FastKing cDNA Kit (Tiangen). The cDNA was diluted 1:10 and served as the template for qPCR analysis using the CFX96 Real-Time PCR Detection system (Bio-Rad) with SYBR Green RealMasterMix (Tiangen) and qPCR primers (Tang et al., 2013). The threshold cycle (C_T_) values for each target gene were normalized with the reference 16S rRNA gene. The 2^ΔΔCT^ method was used to calculate the relative expression level, where ΔΔC_T_ = (C_T_^*target*^ − C_T_^16*S*^)_induction_ − (C_T_^*target*^ − C_T_^16*S*^)_control_ (Livak and Schmittgen, 2001).

## Results and Discussion

*Pseudomonas putida* is a widely used strain for degrading persistent organic compounds. Up to now, no report has been published for enhancing the acid stress resistance for *P. putida*. Applications of a *P. putida* strain to curb environmental pollution under acid stress has therefore been restricted.

### Engineered Strains of *P. putida* S16 and Their Acid Resistance

**FIGURE 1:**
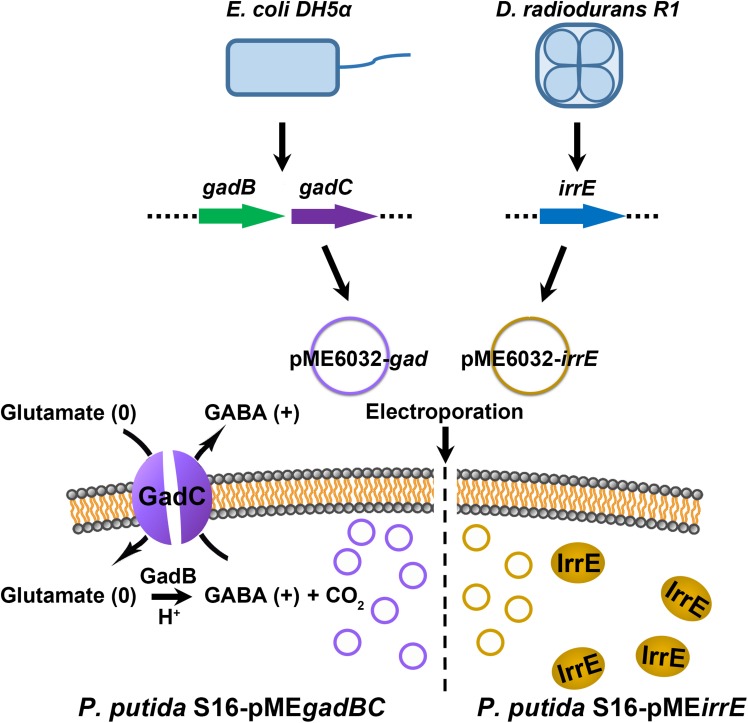
The verification strategy for two different acid resistance systems. The Gad system was obtained from *E. coli* BL21(DE3), and molecular chaperone system was amplified from *D. radiodurans* R1. The plasmid pME6032 was used as empty control or transporter to construct recombinants.

**FIGURE 2:**
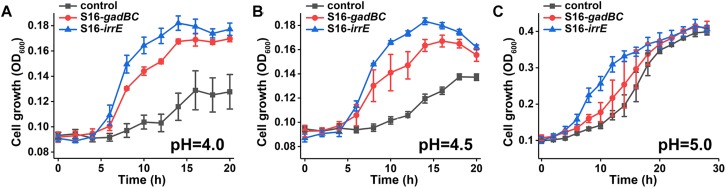
Growth curves in glycerin medium under pH 4.0 **(A)**, pH 4.5 **(B)**, and pH 5.0 **(C)**.

We constructed two different potential acid-resistance devices, *gadBC* and *irrE*, to evaluate the degradation ability of *P. putida* S16 under acidic conditions. The recombinant plasmid pME-Gad constructed by insertion of a 3.3-kb fragment containing the gene *gadBC* and 230 bp upstream from the initiation site of *gadB* is shown in Supplementary Figure S1A, while the recombinant plasmid pME-GirrE holding the gene *irrE* and the promoter GroESL is shown in Supplementary Figure S1C. The possession of the gene expressing gadBC or irrE was confirmed with PCR (Supplementary Figures S1B,D) and DNA sequencing. The overview of the verification strategy if two different acid resistance systems is shown in Figure 1. Over more than 20 h of cultivation, *P. putida* S16 harboring pME-Gad or pME-GirrE displayed better acid tolerance than the control. At pH 4.5 and 4.0, the control bacterium grew much slower compared to the transformant strains with *gadBC* or *irrE* that grew faster and reached to higher cell densities (Figure 2). These results revealed that *gadBC* and *irrE* increased the acid resistance ability in *P. putida* S16, and *irrE* displayed a stronger effect on acid tolerance than *gadBC* in strain S16.

### Pollutant-Degrading Potential of Engineered *P. putida* S16

**FIGURE 3:**
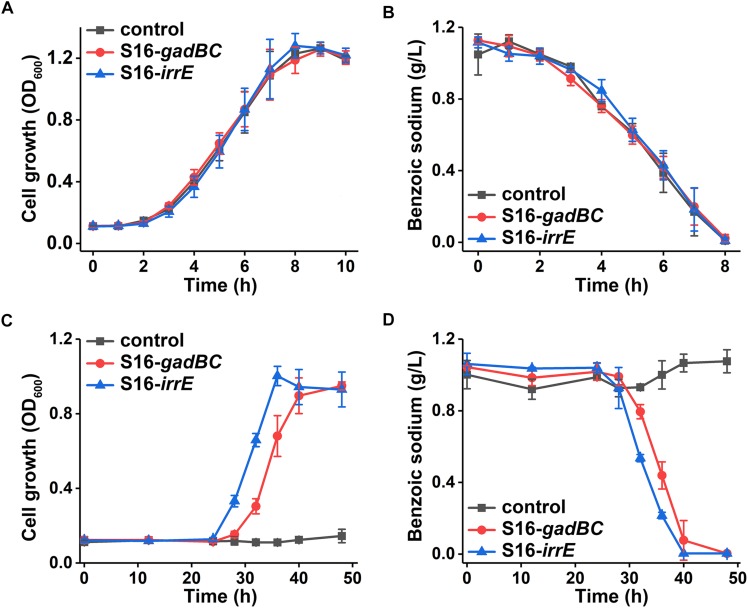
The growth curves in sodium benzoate medium **(A)** and **(C)** and sodium benzoate degradation curves **(B)** and **(D)** in pH 7.0 and pH 5.5, respectively.

Either control or engineered *P. putida* S16 strains displayed similar growth rates and benzoate-degrading efficiencies under pH 7 (Figures 3A,B), which demonstrated that the acid-resistant components did not influence these bacterial strains under neutral conditions. At pH 5.5, either *gadBC* or *irrE* holding strains showed better growth characteristics in comparison to the control. For example, strains with the *gadBC* gene had a maximum growth of 1.0 within 30 h, and strains with the *irrE* gene reached a maximum growth of 0.9 within the same incubation time, whereas no growth was observed for the control. Sodium benzoate degradation rates for *gadBC* and *irrE* were 0.09 and 0.087 g/L/h, respectively (Figures 3C,D). Strains harboring the *irrE* gene have an advantage at early degradation phase, while the ones with the *gadBC* gene had a longer lag phase.

**FIGURE 4:**
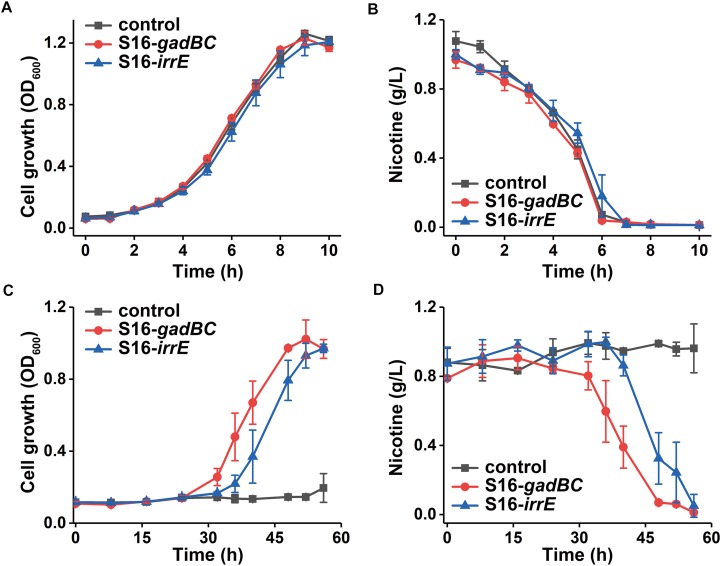
The growth curve in nicotine medium **(A)** and **(C)** and nicotine degradation curve **(B)** and **(D)** under pH 7.0 and pH 5.5, respectively.

Every test strain displayed no difference in either growth rate or nicotine degradation under a neutral environment (Figure 4). However, their growth characteristics differed under an acidic environment, pH 5.5 (Figure 4A). After 56 h of growth, cells harboring pME-Gad or pME-GirrE plasmid reached a maximum growth of 1.0 and grew better than the control. Furthermore, nicotine was entirely degraded by the engineered strains with the *gadBC* or *irrE* gene at pH 5.5 within 60 h, showing the significant acid tolerance compared to the control (Figure 4C). The *gadBC* and *irrE* genes significantly enhanced both the growth rate and degradation efficiency of *P. putida* S16 under acidic conditions.

Based on the results of cell growth (Table 1) and nicotine degradation, both *gadBC* and *irrE* could improve acid-resistance in *P. putida* S16. Under acidic conditions (pH 5.5), both growth and degradation rate of *P. putida* S16 harboring pME-Gad were better than the strain harboring pME-GirrE, and showed a shorter lag phase in nicotine medium. The results were similar to a previous study showing that the GAD system not only contributes to pH homeostasis but, by transiently accumulating GABA in the cell, it counteracts illicit entry of protons by inversion of the membrane potential, a strategy similar to that adopted by extreme acidophiles (Foster, 2004). Past studies of the molecular mechanisms of *gadBC* or *irrE* inducing stress resistance in bacterial cells focused on the enhancement rate of stress resistance ability. In this study, we combined theoretical research with practical environmental degradation to better understand the real affects.

**TABLE 1:** The dry cell weight of *P. putida* S16 harboring different genes at different pH conditions.

**DCW (g/L)**		**pH 7.0**	**pH 5.5**
Benzonic sodium medium	S16-*gadBC*	0.571 ± 0.017	0.418 ± 0.023
	S16-*irrE*	0.602 ± 0.023	0.457 ± 0.039
Nicotine medium	S16-*gadBC*	0.525 ± 0.039	0.446 ± 0.031
	S16-*irrE*	0.534 ± 0.022	0.422 ± 0.034

### Transcriptional Expression of Nicotine-Degrading Genes

**FIGURE 5:**
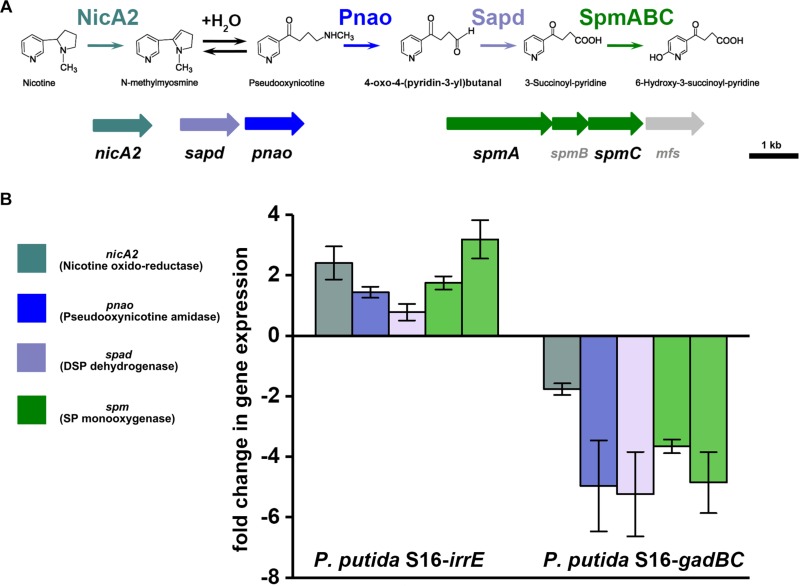
Validation of transcriptional regulation of differentially expressed proteins. RT-qPCR analysis of target gene transcripts produced in *P. putida* S16 harboring *irrE* or GAD systems grown at pH 7 and pH 5.5. **(A)** Nicotine degradation pathway (partial) **(B)** mRNA expression levels of 5 target genes involved in nicotine degradation of *P. putida* S16 were estimated using RT-qPCR and the 2^Δ^ ^Δ^ ^*CT*^ method. The 16S rRNA gene was used as the reference gene. Results presented in these histograms are the means of three independent experiments, and error bars indicate the standard deviations.

To validate the nicotine degradation and assess relative transcription levels of genes related to nicotine degradation in *P. putida* S16 expressing *gadBC* or *irrE*, we utilized RT-qPCR to compare mRNA levels of genes *nicA2*, *pnao*, *sapd*, *spmA*, and *spmC* at pH7 and pH5.5. The RT-qPCR analysis revealed that all target genes were up-regulated in cells harboring pME-GirrE. The *spmC* mRNA expression was 3.2-fold up-regulated which is the highest one in those genes, following were *nicA2* 2.4-fold, *spmA* 1.9-fold, *pnao* 1.7-fold and *sapd* 1.2-fold (Figure 5). On the contrary, in *gadBC* holding strain, those nicotine-degrading genes were down-regulated. These results indicated that the GAD system promoted a different role in nicotine degradation of strain S16 (Figure 5). Combined with the result of growth curves, the strain harboring *irrE* at pH 5.5 had worse growth behavior than the condition of pH 7.0. Interestingly, the entire nicotine degrading genes were upregulated in the strain harboring *irrE* at pH 5.5 at the mRNA level, with the same degradation rate at pH 7.0. We suppose that acidic conditions decreased the optimal enzyme activities, so that the global regulator IrrE has to upregulate expression of nicotine-degrading genes to make up for deficiency of the enzyme activities under acidic conditions.

## Conclusion

Optimization of the GAD system and IrrE expression as tools to promote bacterial growth in acidic environments will be important for further studies. Synthetic biology has shown enormous potential to generate biological modules with unprecedented applications by combining the basic elements of biology (Khalil and Collins, 2010). A more thorough understanding of the acid tolerance mechanisms of microorganisms is needed and, in combination with synthetic biology, may contribute greatly to the industrial processes involving acid-resistant microbes. What’s more, it could not be ignored that horizontal gene transfer would bring unexpected influences for natural microorganisms. Introducing engineered bacteria with mobile genetic elements of strong resistance traits into nature may reach to the increase of resistant pathogens coexisted out there, which is a global threat of antimicrobial resistance problem. For avoiding this risk, we are trying to manipulate engineered bacteria to death using smart sensor modules and suicide modules when the concentrations of pollutants are reduced to normal environmental levels.

## Data Availability

The datasets generated for this study are available on request to the corresponding author.

## Author Contributions

ZZ, PX, and HT conceived the project and wrote the manuscript. ZZ, HT, and YL designed and performed all the experiments. ZZ and YL analyzed the results. ZW and GZ provided samples and materials. GZ revised the manuscript.

## Conflict of Interest Statement

ZW was employed by company SUS Environment Co., Ltd. The remaining authors declare that the research was conducted in the absence of any commercial or financial relationships that could be construed as a potential conflict of interest.

## References

[B1] Chen T. Wang J. Yang R. Li J. Lin M. Lin Z. (2011). Laboratory-evolved mutants of an exogenous global regulator, IrrE from *Deinococcus radiodurans*, enhance stress tolerances of *Escherichia coli*. *PLoS One* 6:e16228. 10.1371/journal.pone.0016228 21267412 PMC3022760

[B2] De Biase D. Pennacchietti E. (2012). Glutamate decarboxylase-dependent acid resistance in orally acquired bacteria: function, distribution and biomedical implications of the *gadBC* operon. *Mol. Microbiol.* 86 770–786. 10.1111/mmi.12020 22995042

[B3] Dixon M. Lambin K. Seeman J. (2000). Mini-review: on the transfer of nicotine from tobacco to the smoke: a brief review of ammonia and “pH” factors. *Beitr. Tabak* 19 103–113. 10.2478/cttr-2013-0700

[B4] Foo K. Y. Hameed B. H. (2009). An overview of landfill leachate treatment via activated carbon adsorption process. *J. Hazard. Mater.* 171 54–60. 10.1016/j.jhazmat.2009.06.038 19577363

[B5] Foster J. W. (2004). *Escherichia coli* acid resistance: tales of an amateur acidophile. *Nat. Rev. Microbiol.* 2 898–907. 10.1038/nrmicro1021 15494746

[B6] Gao G. Tian B. Liu L. Sheng D. Shen B. Hua Y. (2003). Expression of *Deinococcus radiodurans* PprI enhances the radioresistance of *Escherichia coli*. *DNA Repair* 2 1419–1427. 10.1016/j.dnarep.2003.08.012 14642569

[B7] Hu H. Wang L. Wang W. Wu G. Tao F. Xu P. (2019). Regulatory mechanism of nicotine degradation in *Pseudomonas putida*. *mBio* 10 e602–e619. 10.1128/mBio.00602-19 31164460 PMC6550519

[B8] Kanjee U. Houry W. A. (2013). Mechanisms of acid resistance in *Escherichia coli*. *Annu. Rev. Microbiol.* 67 65–81. 10.1146/annurev-micro-092412-155708 23701194

[B9] Khalil A. S. Collins J. J. (2010). Synthetic biology: applications come of age. *Nat. Rev. Genet.* 11 367–379. 10.1038/nrg2775 20395970 PMC2896386

[B10] Kjeldsen P. Barlaz M. A. Rooker A. P. Baun A. Ledin A. Christensen T. H. (2002). Present and long-term composition of MSW landfill leachate: a review. *Crit. Rev. Environ. Sci. Technol.* 32 297–336. 10.1080/10643380290813462

[B11] Li J. Wang J. Li S. Yi F. Xu J. Shu M. (2019). Co-occurrence of functional modules derived from nicotine-degrading gene clusters confers additive effects in *Pseudomonas* sp. *JY-Q*. *Appl. Microbiol. Biotechnol.* 103 4499–4510. 10.1007/s00253-019-09800-4 31016356

[B12] Lin Z. Zhang Y. Wang J. (2013). Engineering of transcriptional regulators enhances microbial stress tolerance. *Biotechnol. Adv.* 31 986–991. 10.1016/j.biotechadv.2013.02.010 23473970

[B13] Livak K. Schmittgen T. (2001). Analysis of relative gene expression data using real-time quantitative PCR and the 2^−ΔΔ*C*_T_^ method. *Methods* 25 402–408. 10.1006/meth.2001.1262 11846609

[B14] Ma D. Lu P. Yan C. Fan C. Yin P. Wang J. (2012). Structure and mechanism of a glutamate-GABA antiporter. *Nature* 483 632–636. 10.1038/nature10917 22407317

[B15] Ma R. Zhang Y. Hong H. Lu W. Lin M. Chen M. (2011). Improved osmotic tolerance and ethanol production of ethanologenic *Escherichia coli* by IrrE, a global regulator of radiation-resistance of *Deinococcus radiodurans*. *Curr. Microbiol.* 62 659–664. 10.1007/s00284-010-9759-2 20959988

[B16] Oie C. S. Albaugh C. E. Peyton B. M. (2007). Benzoate and salicylate degradation by *Halomonas campisalis*, an alkaliphilic and moderately halophilic microorganism. *Water Res.* 41 1235–1242. 10.1016/j.watres.2006.12.029 17292440

[B17] Pan J. Wang J. Zhou Z. Yan Y. Zhang W. Lu W. (2009). IrrE, a global regulator of extreme radiation resistance in *Deinococcus radiodurans*, enhances salt tolerance in *Escherichia coli* and *Brassica napus*. *PLoS One* 4:e4422. 10.1371/journal.pone.0004422 19204796 PMC2635966

[B18] Su M. S. Schlicht S. Ganzle M. G. (2011). Contribution of glutamate decarboxylase in *Lactobacillus reuteri* to acid resistance and persistence in sourdough fermentation. *Microb. Cell Fact.* 10:S8. 10.1186/1475-2859-10-S1-S8 21995488 PMC3231934

[B19] Tang H. Wang L. Wang W. Yu H. Zhang K. Yao Y. (2013). Systematic unraveling of the unsolved pathway of nicotine degradation in *Pseudomonas*. *PLoS Genetics* 9:e1003923. 10.1371/journal.pgen.1003923 24204321 PMC3812094

[B20] Wang S. Liu Z. Tang H. Meng J. Xu P. (2007). Characterization of environmentally friendly nicotine degradation by *Pseudomonas putida* biotype A strain S16. *Microbiology SGM* 153 1556–1565. 10.1099/mic.0.2006/005223-0 17464070

[B21] Wang S. Xu P. Tang H. Meng J. Liu X. Huang J. (2004). Biodegradation and detoxification of nicotine in tobacco solid waste by a *Pseudomonas* sp. *Biotechnol. Lett.* 26 1493–1496. 10.1023/b:bile.0000044450.16235.65 15604785

[B22] Xie N. Tang H. Feng J. Tao F. Ma C. Xu P. (2009). Characterization of benzoate degradation by newly isolated bacterium *Pseudomonas* sp. XP-M2. *Biochem. Eng. J.* 46 79–82. 28124304

[B23] Yu H. Tang H. Wang L. Yao Y. Wu G. Xu P. (2011). Complete genome sequence of the nicotine-degrading *Pseudomonas putida* strain S16. *J. Bacteriol.* 193 5541–5542. 10.1128/JB.05663-11 21914868 PMC3187465

[B24] Zhong W. Zhu C. Shu M. Sun K. Zhao L. Wang C. (2010). Degradation of nicotine in tobacco waste extract by newly isolated *Pseudomonas* sp. ZUTSKD. *Bioresour. Technol.* 101 6935–6941. 10.1016/j.biortech.2010.03.142 20434329

