# Supplementary material for: Enhancing Bioremediation Potential of Pseudomonas putida by Developing Its Acid Stress Tolerance With Glutamate Decarboxylase Dependent System and Global Regulator of Extreme Radiation Resistance
Source: Front Microbiol. 2019 Sep 4;10:2033. doi: 10.3389/fmicb.2019.02033 (PMC6738132; doi:10.3389/fmicb.2019.02033)
Supplement: Supplementary file 1 [file Data_Sheet_1.docx]

Supplementary Material


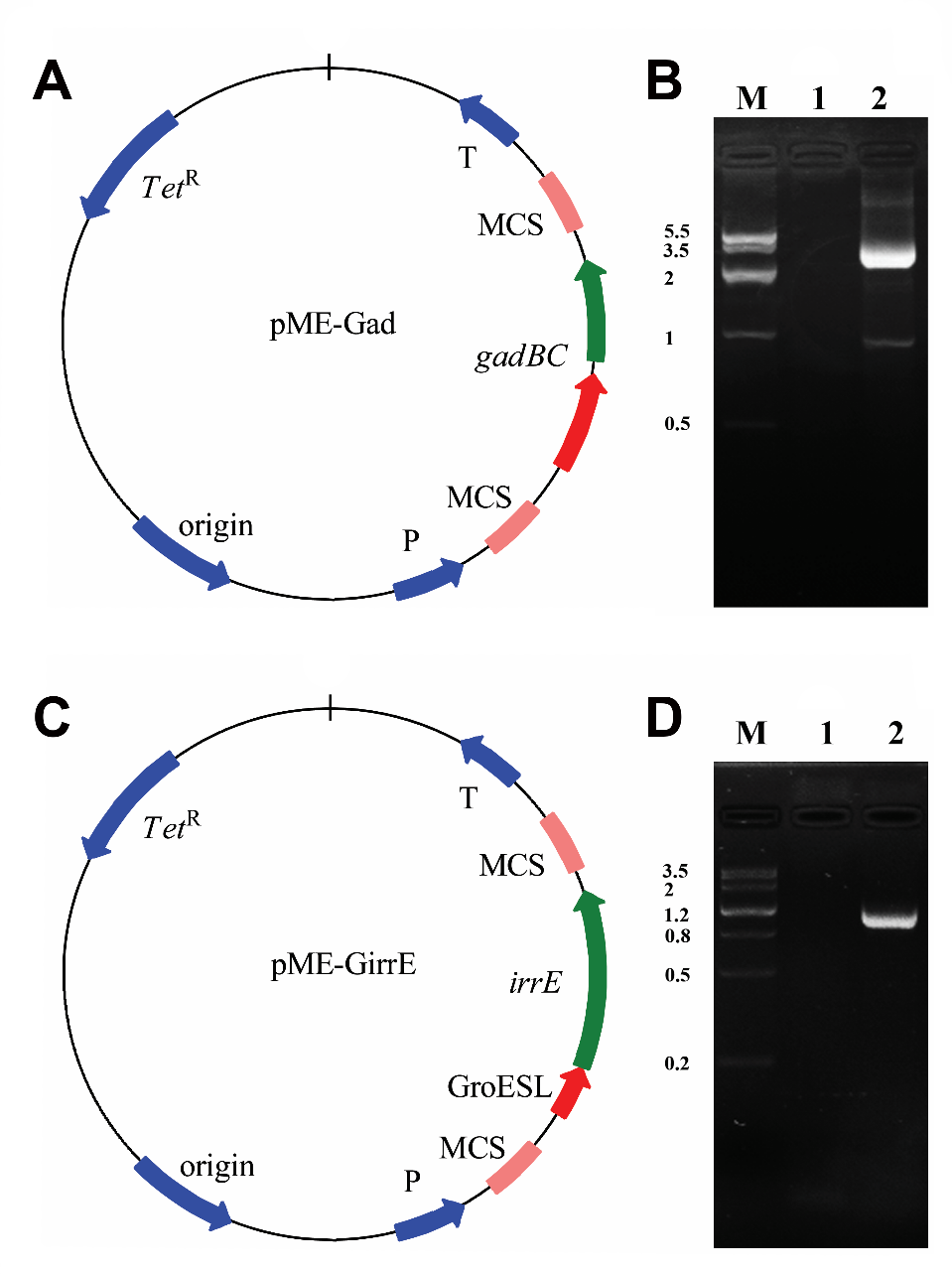


**Figure S1.** The recombinant construction. **A**. and **C**. The recombinant plasmid pME-Gad (plasmid pME6032 inserted with *gadBC*) and the recombinant plasmid pME-GirrE (plasmid pME6032 inserted with GroESL-*irrE*). (‘-’ represents wild type *P. putida* S16, ‘*gadBC*’ represents the *gadBC* expressing strains and ‘*irrE*’ represents the *irrE* expressing strains). **B**. ad **D**. Verification of plasmid pME-Gad and pME-GirrE transformation

(‘-’ represents wild type *P. putida* S16, ‘*gadBC*’ represents the *gadBC* expressing strains and ‘*irrE*’ represents the *irrE* expressing strains).


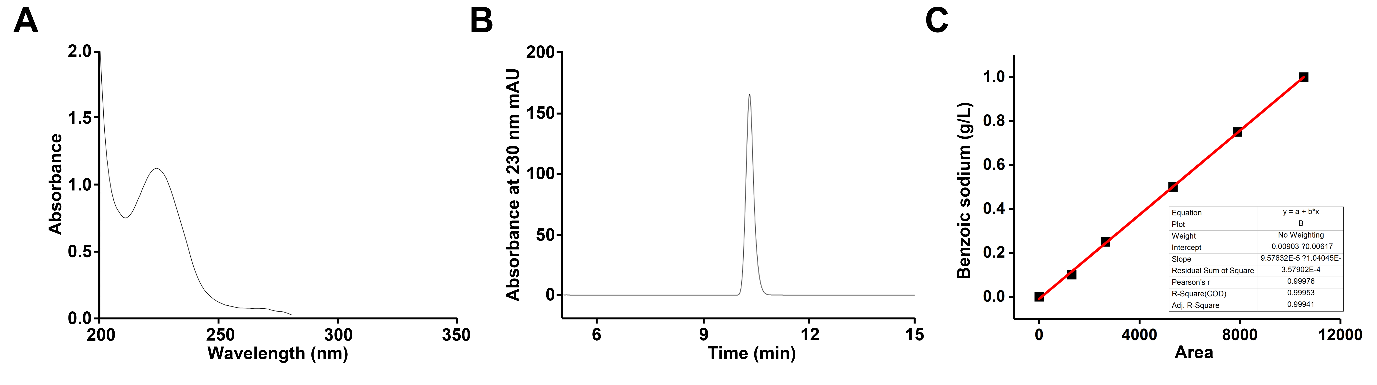


**Figure. S2** The standard curve of benzoic sodium. **A**. the full wavelength scanning shows benzoic sodium absorbance peak at 230 nm. **B**. HPLC spectrum of standard sample of benzoic sodim. **C**. the standard curve with R^2^ = 0.99953, performed by Origin 8.5.


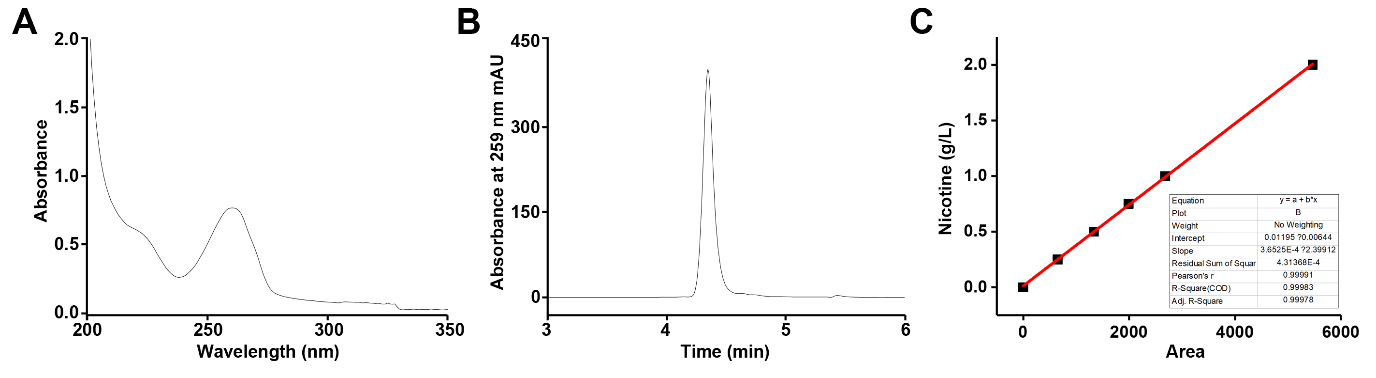


**Figure. S3** The standard curve of nicotine. **A**. the full wavelength scanning shows nicotine absorbance peak at 259 nm. **B**. HPLC spectrum of standard sample of nicotine. **C**. the standard curve with R^2^ = 0.99983, performed by Origin 8.5
